# Investigating the Effect of Smoke Treatment on Hygroscopic Characteristics of Bamboo by FTIR and Raman Spectroscopy

**DOI:** 10.3390/ma15041544

**Published:** 2022-02-18

**Authors:** Raviduth Ramful, Thefye P. M. Sunthar, Elia Marin, Wenliang Zhu, Giuseppe Pezzotti

**Affiliations:** 1 Graduate School of Science and Technology, Kyoto Institute of Technology (KIT), Kyoto 606-8585, Japan; 2 Mechanical and Production Engineering Department, Faculty of Engineering, University of Mauritius, Reduit 80837, Mauritius; 3 Ceramic Physics Laboratory, Kyoto Institute of Technology, Kyoto 606-8585, Japan; d0871502@edu.kit.ac.jp (T.P.M.S.); elia-marin@kit.ac.jp (E.M.); wlzhu@kit.ac.jp (W.Z.); pezzotti@kit.ac.jp (G.P.); 4 Department of Immunology, Graduate School of Medical Science, Kyoto Prefectural University of Medicine, Kamigyo-ku, Kyoto 602-0841, Japan; 5 Department of Dental Medicine, Graduate School of Medical Science, Kyoto Prefectural University of Medicine, Kyoto 602-8566, Japan; 6 The Center for Advanced Medical Engineering and Informatics, Osaka University, Osaka 565-0871, Japan; 7 Department of Orthopedic Surgery, Tokyo Medical University, Tokyo 105-8461, Japan

**Keywords:** bamboo, smoke treatment, hygroscopicity, durability, Raman spectroscopy

## Abstract

Treatment modification to improve the durability of bamboo against biotic and abiotic factors often results in adverse effects to its mechanical properties due to changes in hygroscopic characteristics. This study aims at exploring in more detail, the effect of treatment modification, in particular smoke treatment, on the hygroscopic nature of bamboo. In the first part of this study, changes to its chemical structure were investigated by Raman and Fourier-transform infrared (FTIR) spectroscopic techniques. From Raman analysis, specific bands attributed to lignin component in bamboo, namely at 1600 cm^−1^ and 1632 cm^−1^, which varied in intensities among treated and untreated specimens, could be considered to assess the extent of treatment modification. Besides, FTIR results showed that the chemical constituents of bamboo inner and outermost surfaces vary extensively with distinctive changes during treatment modification. The steam component in smoke treatment is assumed to cause a slight increase in the moisture content in the outermost surface of smoked bamboo as evidenced by FTIR results. In addition, the hydrophobic surface of smoked bamboo, which was affected during smoke treatment modification due to superior mean roughness parameter in its outermost surface, impacted its water-repelling ability. From FTIR results, an increase in lignin in bamboo was confirmed at peak 1114 cm^−1^, which occurred as a result of thermal effect above a temperature of 100 °C leading to poly-condensation reactions. The increase in lignin is assumed to cause an overall increase in hardness of smoked bamboo which was found to be two-fold higher when compared with the untreated ones. The approach of this research investigation, which has shown the benefit of using spectroscopic techniques to monitor and understand the changes in the hygroscopic nature of bamboo surfaces, can likewise be considered to predict the corresponding effects of treatment modification or degradation on the mechanical properties of natural materials.

## 1. Introduction

Bamboo surpasses the structural limitations experienced by most natural composite materials and displays notable attributes which make it a suitable material for construction. The main hindrance encountered by most natural materials, namely in terms of their limited durability, greatly impacts the full commercialization of bamboo-based constructions. Environmental factors such as ultraviolet radiation, rain, and humidity are among the few abiotic factors which affect the durability and structural performance of bamboo. Besides, the hygroscopicity trait, which is the affinity of bamboo to absorb water content via its surface, significantly influences the premature fracture in bamboo materials due to non-uniform shrinkage and swelling [1]. One way to improve its durability limitations is via treatment modification such as natural drying which enhances its dimensional stability and its resistance to natural degradation [2,3,4]. In the recent past, traditional smoke treatment, which have been used to modify bamboo culms for use in construction housing in Asian and South-American countries, have shown excellent longevity in terms of both performance and durability [5,6].

The numerous benefits found in smoke-treated bamboo is presumed to lie in its treatment methodology. The heat in smoke treatment led to partial pyrolysis of bamboo outer surface at a temperature of about 120 °C. This temperature increase was enough to alter the structure of lignin, hence leading to poly-condensation reactions and further cross-linking of lignin networks [7]. The higher percentage of lignin content in smoked bamboo thus contributed to its improved dimensional stability and higher strength namely in its outermost layers, which were subjected to smoke treatment [5]. Besides, its resistance to degradation against insect attack was achieved by the effect of heat and soot generated during pyrolysis which led to a reduction in the starch content. At elevated temperature, the de-polymerization of carbohydrates is also assumed to contribute to this effect [2,5]. This traditional treatment is a proven eco-friendly alternative to conventional chemical treatments and is highly regarded due to their effectiveness at enhancing bamboo durability against hygrothermal effects and resistance against degradation by fungal decay and termite attack [3,4]. The treatment has been modified for large-scale production of smoked bamboo culms in Colombia whereby semi-dry culms are subjected to a combustion smoke of organic matter at a temperature of 55 °C inside furnace environment for a period of 15–30 days. This process will reduce the moisture content to about 12% as bamboo culms undergo partial carbonization [2,3]. The heat treatment also led to a reduction in starch as the parenchyma cells were found to be damaged by the effect of heat [4].

In order to investigate and understand the mechanisms emerging from surface modification such as smoke treatment, it is important to assess the change in the main cellular constituents of bamboo composed of cellulose, hemicellulose, and lignin [4,5,6]. Recent improvement to non-invasive and non-destructive spectroscopic techniques such as the Fourier-transform infrared spectroscopy (FTIR) and Raman spectroscopy enables precise and remote monitoring of real-time molecular changes in natural materials [8,9,10]. Fourier transform (FT)-Raman spectroscopy has been used in numerous studies to provide complementary information about the chemical constituents, functional groups, and changes due to treatment modification in wood and bamboo materials [8,11,12,13]. It has been widely applied to determine changes in the chemical constituents of carbonized bamboo through crystallinity analysis [14,15,16,17]. Furthermore, confocal Raman microscopy technique, which can provide high resolution images, has been used to reveal cell wall chemistry at micro-level such as the determination of microfibril angle and degree of lignification in cell wall components [18,19]. Changes to the chemical structure of plant cell wall polymers at sub-micron level, such as lignification of plant tissues and changes in cellulose content, have been revealed via the Raman microspectroscopy technique [9]. The ratio of normalized heights of the absorption peaks of control and treated bamboo samples were found to be useful to assess changes due to treatment modification [20]. FTIR has also been used in various studies to assess the chemical change due to treatment modification in the surface of bamboo structure [1,6].

Besides the long-term durability of smoked bamboo to degradation resistance, the treatment tends to significantly affect its physical and mechanical properties, namely its hygroscopicity, ductility, and toughness, hence increasing its susceptibility to split. The aim of this study is to probe into the effect of smoke treatment on the hygroscopic characteristic of smoke-modified bamboo by first investigating the alterations to its chemical structure and second, changes to its interface characteristics and mechanical properties. In first part of this study, spectroscopic techniques, namely FTIR and Raman spectroscopy were considered to investigate the mechanisms due to surface modification. Key findings in terms of chemical changes discerned on the surface of modified specimens through spectroscopy techniques, were further verified by additional surface analysis, namely by surface roughness, contact angle, and hardness tests.

The results of this study will be useful to qualitatively assess the relative changes in the physical and mechanical properties of naturally treated bamboo via the non-invasive evaluation method of spectroscopy analysis. In terms of significance, the results could be applied to other natural materials like wood to estimate the corresponding effect of treatment modification or natural degradation on their mechanical properties by considering a similar approach involving spectroscopy techniques.

## 2. Materials and Methods

### 2.1. Specimen Preparation

**Figure 1:**
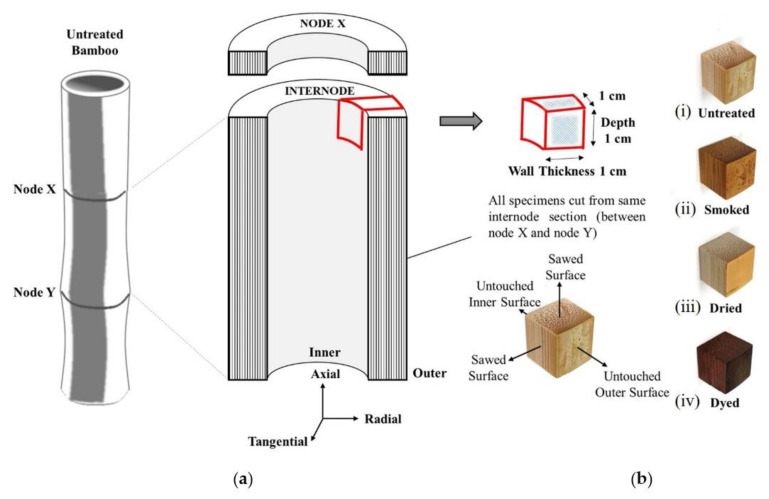
(**a**) Schematic illustration of specimen sizing; (**b**) types of bamboo used in this study: (i) untreated, (ii) smoked, (iii) naturally dried, and (iv) dyed.

In this study, four categories of widely used bamboo materials in Japan were investigated, namely natural, dried, dyed, and smoked bamboo. All four categories of treated specimens were procured from Yokoyama Bamboo Products & Co. in Kyoto, Japan (35.02457769543427, 135.75385594905308). The raw bamboo had a maturity of 3 years and were harvested in autumn from Kameoka in the eastern region of Kyoto, Japan. To minimize inconsistency in test results, all bamboo specimens used in this research investigation were prepared from a single culm from the species of Madake Bamboo (Phyllostachys bambusoides) as displayed in Figure 1a. The test-ready specimens were sized in cubed shape of 1 cm in length by taking into account their full wall thickness, which had dimensions of 1 cm. The actual representation of various bamboo samples prepared from raw bamboo culm is shown in Figure 1b.

In traditional housing construction in Japan, the long-term durability of smoked bamboo culm was acquired in a slow and continuous combustion smoke treatment which was mainly derived from organic matter. This slow-treatment process was previously replicated by subjecting natural bamboo to a combustion smoke of organic matter for a period of 15–30 days until the moisture content was reduced to about 12% [2]. In this study, a modified smoke treatment was used to replicate the partial carbonization process observed in smoked bamboo by considering an accelerated 24-h treatment period inside a furnace environment set at a maximum temperature of 150 °C. The smoke treatment was composed of a mixture of steam and combustion smoke of organic matter involving wood and bamboo. The resulting brown coloration in smoked specimen occurred as a result of the soot deposition, which is a black powdery substance produced during the incomplete combustion of organic matter.

**Figure 2:**
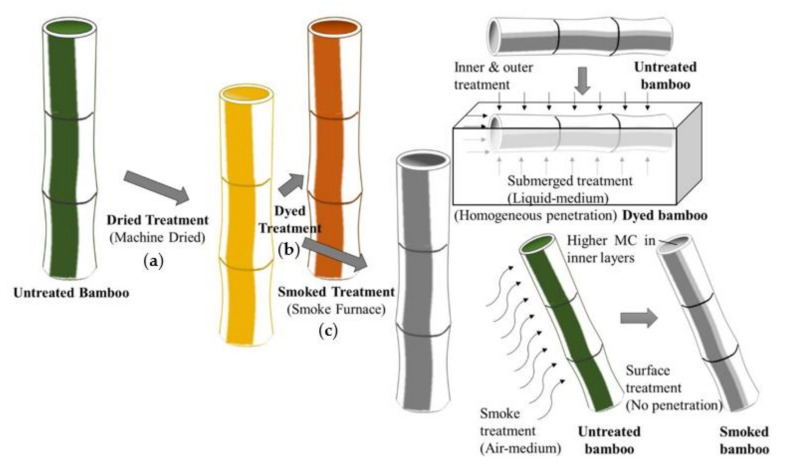
Schematic illustration of methodology of specimen preparation considered in this study; (**a**) dried treatment, (**b**) dyed treatment and (**c**) smoked treatment.

For the purpose of comparing the characteristics of smoke-modified bamboo, another two types of treatment-modified samples, namely dried and dyed, which differed in treatment methodologies, were considered. To minimize the incidence of cracking, dried bamboo is generally prepared from harvested bamboo by natural drying in forest in the first two months following harvest and an additional 12-month inside in factory environment. In this study however, since all specimens were prepared from the same culm, the dried samples were prepared from natural bamboo by kiln oven drying method followed by a heat treatment to remove oil from its hard, waxy outer surface. Dyed specimens were obtained by subjecting dried bamboo to a submerged chemical-bath treatment consisting of colored pigments (Dianix blue E-GR and Miketon polyester yellow, DyStar, Osaka, Japan). In this study, natural bamboo specimens, prepared from untreated Madake Bamboo, were used as control. The schematic illustration of treatment methodologies used to prepare various bamboo samples are outlined in Figure 2.

### 2.2. Spectroscopic Analysis

In the first spectroscopy technique, Raman spectra were collected using a dedicated spectroscope (T-64000, Horiba/Jobin-Yvon, Kyoto, Japan). The spectroscope was equipped with a CCD (charged coupled device) detector and operated in microscopic confocal mode with a 100× optical lens with a limited probe area of about 1 μm. For high-resolution spectral acquisitions (greater than 0.1 cm^−1^), a triple-monochromator was used. The excitation source used was a laser at an excitation wavelength of 514.532 nm. The lateral movement of the stage was controlled with two high precision step motors with a resolution in the order of 1 μm. An average of six spectra were collected on each of the four different category of bamboo specimens. The spectra were then analyzed by commercially available software (LabSpec, Horiba, Kyoto, Japan and Origin, Originlab Corp., Northampton, MA, USA).

In the second spectroscopy technique, the effect of treatment modifications on the cellular constituents of bamboo was assessed by FTIR. Based on previous study, FTIR was found to be a complementary analytical technique which could provide useful information, for comparison purposes, about the chemical change between untreated and modified specimens [21]. FTIR analysis was conducted via attenuated total reflection Fourier transform infrared spectroscopy (ATR-FTIR, FTIR-4700 with ATR PRO ONE fitted with a diamond prism; Jasco Co., Tokyo, Japan). FTIR measurements were performed between the range of 400 and 4000 cm^−1^. A resolution of 4 cm^−1^ and including 100 scans were considered throughout the FTIR analysis. Commercial software, namely LabSpec (Horiba/Jobin-Yvon, Kyoto, Japan) and Origin 8.5 (OriginLab Co., Northampton, MA, USA) were used for spectral acquisition and pre-processing of raw data by baseline subtraction, smoothing, normalization, and fitting methods respectively.

### 2.3. Laser Microscopy and Roughness Measurement

Effect of treatment modification on surface morphology of bamboo was characterized at microscopic level by using a confocal scanning laser microscope capable of high-resolution images (Keyence VK-X200 series Laser Microscope, Osaka, Japan). The laser microscope was operated with an automated x-y stage while the *z*-axis was fitted with an autofocus function. Maps collected at magnification of 10× and at numerical aperture of 0.3 were assembled and analyzed in the VK Analyzer (Keyence Corporation, Osaka, Japan).

Roughness was calculated from results of laser microscope by using the multi-line roughness analysis method on the MultiFileAnalyzer (Keyence Corporation, Osaka, Japan). The mean roughness parameter Ra and the average maximum peak to depth parameter Rz were calculated.

### 2.4. Contact Angle Measurement

The contact angle, which is a surface characterization parameter, was based on the measurement of pure liquids on the surface of solids to obtain the surface energy in solid materials. In this study, the contact angle of four main surfaces of bamboo specimen, namely cross-sectional, outermost, innermost, and longitudinal-radial surfaces, were measured by a Phoenix 300 contact-angle system (Kromtek Co., Selangor, Malaysia) at 20 °C. Five microliters of deionized water was dropped onto the various investigated surfaces of bamboo. The average water contact angle was obtained from different locations and further measured by Imagej software (Rasband, W.S., ImageJ, National Institutes of Health, Bethesda, MD, USA).

### 2.5. Hardness Test

Finally, to check the influence of treatment modification on mechanical properties, hardness test was conducted via the Shimadzu HMV Micro Hardness Tester (Shimadzu Corporation, Kyoto, Japan). The longitudinal-radial section of bamboo specimen was indented using the Vickers hardness tester with an HV1 force setting of 9.807 N and retention time set at 15 s. The indented surface was then observed under the Keyence VK-X200 series Laser Microscope (Keyence, Osaka, Japan) in order to accurately measure the length of indented diagonals d1 and d2. Each specimen was indented three times and the average length values of diagonals d1 and d2 were calculated.

### 2.6. Statistical Analysis

Statistical test in terms of an unpaired *t*-test with a significance level of 0.05 was conducted to verify the significance of difference prevailing between specimens in various tests, namely in mean roughness, contact angle, and hardness tests. Statistically significant and insignificant differences were termed and displayed on each diagram with an asterisk “*” and with a not-significant “NS” labels respectively.

## 3. Experimental Results

### 3.1. Raman Spectroscopy Analysis

**Figure 3:**
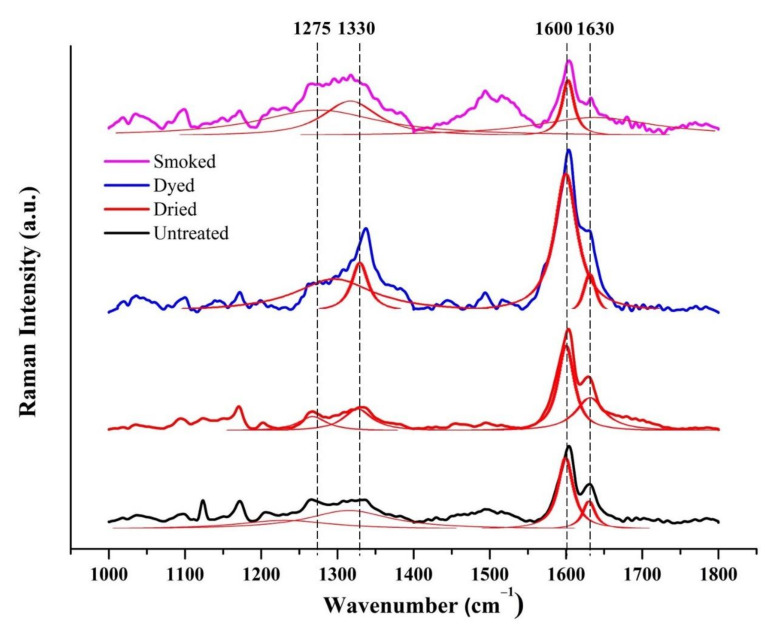
The averaged, normalized, and deconvoluted Raman spectra of untreated and modified bamboo specimens in the spectral range of 1000–1800 cm^−1^.

The averaged, normalized, and deconvoluted Raman spectra of untreated and modified bamboo specimens in the 1000–1800 cm^−1^ spectral range are shown in Figure 3. In the normalization stage, the specific band in the spectral range of 1156 cm^−1^ and 1180 cm^−1^ corresponding to peak intensity of 1173 cm^−1^, which displayed approximately same intensities in all four specimens, was considered as reference band. All Raman spectra were normalized by considering peak intensity of 1173 cm^−1^ as reference band. The spectral deconvolution results were obtained through the peak searching and fitting method in LabSpec by using the GaussLoren function setting. The sub-band fitting at selected peaks in the Raman spectra were automatically conducted in LabSpec until satisfactory results with low error was obtained. The deconvoluted sub bands of the Raman spectra are represented in red.

Distinct bands at 1330 cm^−1^ and 1630 cm^−1^, as observed in the analyzed Raman spectra of all specimens in Figure 3, have previously been observed in the Raman spectra of raw bamboo [14]. These bands at 1330 cm^−1^ and 1630 cm^−1^ have been reported to correspond to the characteristic peaks of hemicellulose and lignin in bamboo respectively [19]. The band range between 1525 cm^−1^ and 1750 cm^−1^ was attributed to lignin mainly present in cell walls of bamboo fibers [18]. The bands at 1330 cm^−1^ and 1630 cm^−1^ were also observed in carbonized bamboo material which were assigned to D and G bands of graphitic carbon respectively [14,15,16]. The intense peak at 1630 cm^−1^ in smoked bamboo, which was also reported in bamboo-based carbon filament, was assigned to the C=C bond vibration in lignin component of bamboo [17].

The Raman spectra, which differed due to the different chemical changes in bamboo as a result of treatment modification, were further analyzed by considering the ratio of normalized peaks. From Table 1, peak intensities at 1600 cm^−1^ and 1632 cm^−1^, which have been attributed to lignin component in bamboo [8,18,19], with respect to normalized peak intensity of 1173 cm^−1^ gives an accurate indication of the relative changes among modified bamboo specimens. The lignin-specific peaks of varying intensities could be considered to reveal the pronounced differences which prevail in various bamboo specimens as a result of treatment modification. In this context, the Raman band ratio (I_1600_/I_1173_) was used as an indicator to assess the relative change of treatment modification with respect to untreated bamboo. In untreated and dried bamboo, the peaks at 1600 cm^−1^, which corresponds to strong aryl ring stretching of lignin in cell corner of bamboo fibers, was two-fold greater than the second of the doublet peak at 1632 cm^−1^ [18]. Besides, the doublet peak at 1600 cm^−1^ and 1632 cm^−1^ also gives an indication of the presence of ferulic and *p*-coumaric acids in bamboo cell walls [5,8,13,18]. Both I_1600_/I_1173_ and I_1632_/I_1173_ ratios clearly show a decreasing trend in peak intensities of smoked bamboo in comparison to the untreated and dried ones. The notable increase in both peaks in dyed bamboo is attributed to the specific changes caused by the chemical compound present in the chemical dye.

### 3.2. FTIR Analysis

**Figure 4:**
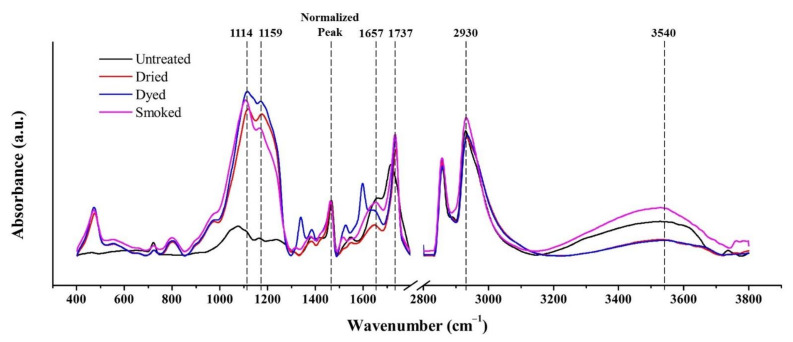
FTIR spectra in the range of 400–1800 cm^−1^ and 2800–3800 cm^−1^, obtained from the outermost layers of untreated and modified bamboo specimens.

The results of FTIR spectra taken in the outside wall section of bamboo specimen in the range of 400–1800 cm^−1^ and 2800–3800 cm^−1^ are displayed in Figure 4. Characteristic bands of FTIR spectra in the frequency interval of 400 to 1800 cm^−1^ and 2800 to 3800 cm^−1^ obtained from the outside surfaces of bamboo samples are given in Table 2.

At peak 1114 cm^−1^, the major increase in the FTIR spectra of dried, dyed, and smoked specimens was attributed to the C-H functional group in guaiacyl and syringyl (lignin) [22]. The notable increase at this band in dried specimen is due to the apparent effect of oxidation which took place during the drying process inside in furnace environment. The trend is same in dyed and smoked bamboo which were derived from dried bamboo. The decrease in the peak at 1657 cm^−1^ which is significant in smoked and dried bamboo in comparison to green bamboo, corresponds to a reduction in their hygroscopicity [23]. Reduction in hygroscopicity also occurred during the formation of new alcohols and esters linked to lignin at band 1114 cm^−1^ which decreased the number of free hydroxyl groups [22]. Moreover, the increase in the peak at 1737 cm^−1^ in smoked and dried bamboo, substantiate the increase of carbonyl groups in lignin under oxidizing conditions [24].

In the spectral range of 2800–3800 cm^−1^, the two peaks observed at 2930 cm^−1^ and at 3540 cm^−1^ were assigned to C-H and O-H functional groups respectively. The increase in peak of smoked specimen relative to untreated bamboo at 3540 cm^−1^, occurred as a result of the nature of smoked treatment which was composed of a mixture of steam and combustion smoke of organic matter [22,25].

**Figure 5:**
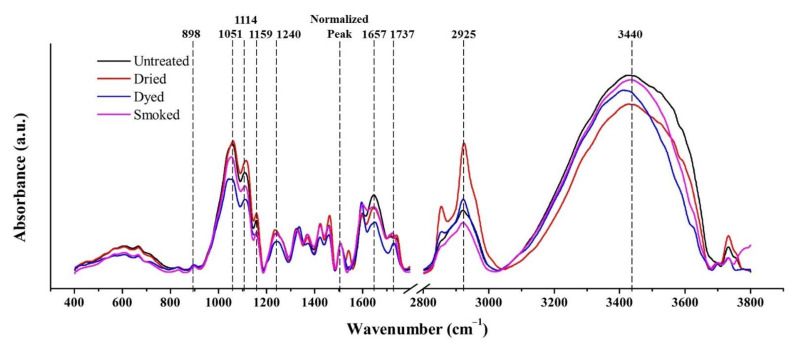
FTIR spectra in the range of 400–1800 cm^−1^ and 2800–3800 cm^−1^, obtained from the innermost layers of untreated and modified bamboo specimens.

The former FTIR results were compared with the ones of FTIR spectra taken in the inside wall section of bamboo specimen in the range of 400–1800 cm^−1^ and 2800–3800 cm^−1^ as shown in Figure 5. The disparity which was observed at peak 1114 cm^−1^, between treated and untreated specimens were not obvious in FTIR results taken from the inside wall section. It could thus be concluded that less oxidation took place inside the inner portion of bamboo culm section.

Similar observations were made in the spectral range of 2800–3800 cm^−1^, as in comparison to FTIR results taken from the outside wall section, no significant disparity between treated and untreated specimens was observed at peak 3540 cm^−1^, which was associated to C-H functional group as per the assignment in Table 2. The inner section of bamboo culm was thus confirmed to be less affected by the steam component of smoke treatment.

### 3.3. Laser Microscopy and Roughness Analysis

**Figure 6:**
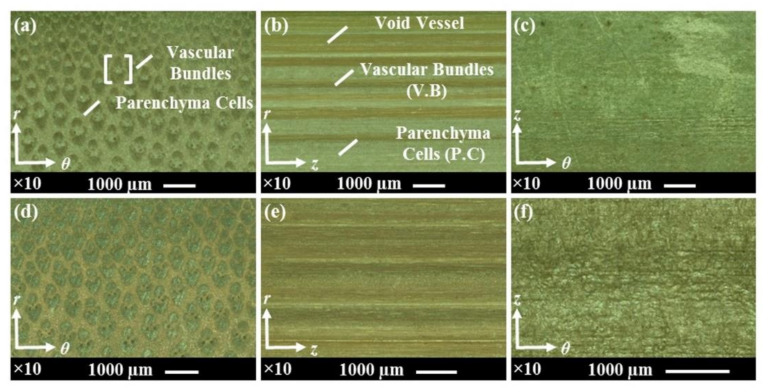
Laser micrographs of radial-tangential, longitudinal-radial and longitudinal-tangential surfaces of: (**a**–**c**) untreated bamboo and (**d**–**f**) smoked bamboo.

The laser micrographs of radial-tangential, longitudinal-radial, and longitudinal-tangential surfaces of untreated and smoked bamboo, obtained at a magnification of ×10, are shown in Figure 6. The distribution of vascular bundles across the thickness was constant as specimens were sized from the same internode section. In comparison to untreated bamboo, some textural changes could be discerned in the radial-tangential and longitudinal-radial surfaces of smoked bamboo. These changes were further assessed by conducting a roughness analysis of these surfaces.

**Figure 7:**
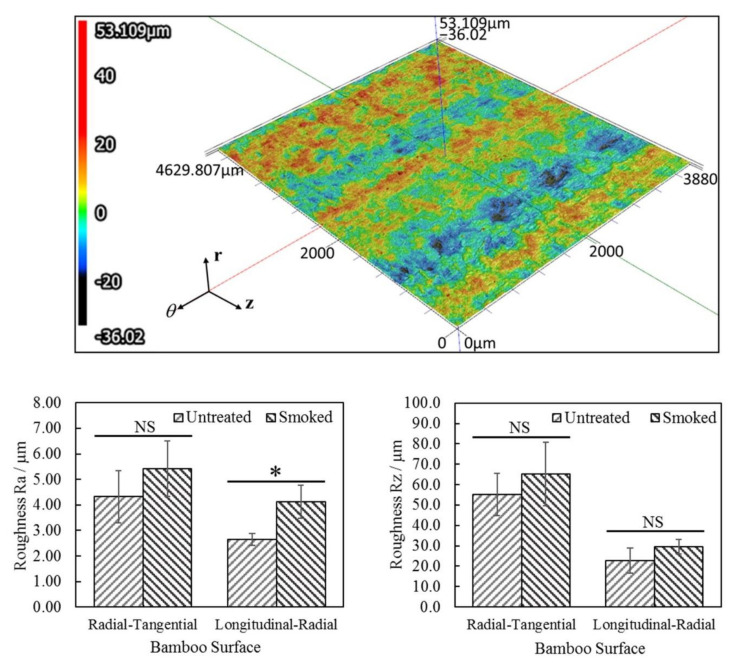
(**a**) 3D map of longitudinal-radial surface of smoked bamboo with a height magnification of 100% and (**b**) comparative study of mean roughness parameter Ra and (**c**) of the average maximum peak to depth parameter Rz (* statistically significant difference, NS = no significant difference).

Figure 7a shows the 3D map with a height magnification of 100% which has been processed for surface shape correction by wave form removal prior to roughness analysis. Roughness analysis of the radial-tangential and longitudinal-radial surfaces of untreated and smoked bamboo was conducted by considering the mean roughness parameter Ra and the average maximum peak to depth parameter Rz. The parameters Ra and Rz were obtained by the multi-line roughness analysis method based on the data collected from five locations in each sample via the laser profilometry technique. The results of roughness analysis on bamboo specimens are displayed in Figure 7b,c. From roughness analysis of Figure 7a, the difference observed in the mean roughness parameter Ra prevailing between the longitudinal-radial surface of untreated and smoked bamboo was found to be statistically significant (*p* > 0.05). In contrast, no statistically significant difference was observed in the mean roughness parameter Ra involving radial-tangential surfaces since both surfaces were manually sawed ones. In terms of the average maximum peak to depth parameter Rz, no statistically significant differences were observed between the radial-tangential surfaces and between the longitudinal-radial surfaces of untreated and smoked bamboo.

**Figure 8:**
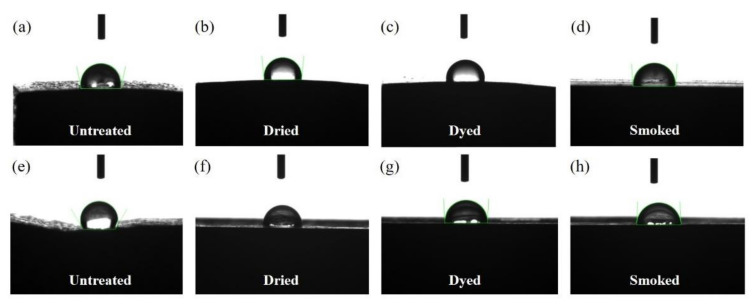
Contact angle measurements taken from (**a**–**d**) the outer and (**e**–**h**) inner sections of untreated and modified bamboo specimens by the Phoenix 300 contact-angle system; the contact angle was calculated by using the Imagej software.

Contact angle was measured by considering the angle between the droplet of deionized water and the outer and inner sections of untreated and modified bamboo specimens as illustrated Figure 8. Both the cross-sectional and longitudinal-radial surfaces readily absorbed the five microliters of deionized water and were therefore categorized as highly hydrophilic and porous surface.

### 3.4. Contact Angle Measurement Results

**Figure 9:**
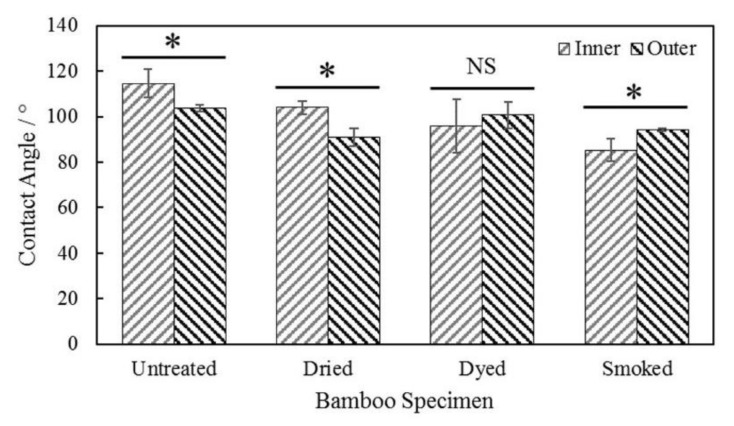
Water contact angle measurements on the inner and outer longitudinal tangential surfaces of untreated and modified bamboo specimens (* statistically significant difference, NS = no significant difference).

The contact angle measurement results of the outermost and innermost surfaces are given in Figure 9. A decreasing trend was observed in the contact angle of outermost and innermost surfaces of both dried and smoked bamboo in comparison to the untreated ones. Smoked bamboo displayed an overall lowest contact angle in its outermost and innermost layers at around 94° and 85° respectively. This marked change, particularly on the outermost surface of bamboo, occurred as a result of the combined effect of thermal modification and steam component present during smoke treatment.

Table 3 shows the results of statistical test performed between treated and untreated specimens as well as between the results collected from the inner and outer wall section in each specimen. In comparison to the contact angle results of inner wall section of untreated bamboo, statistically significant difference (*p* < 0.05) was observed in one smoked bamboo only. Second, significant difference with respect to the contact angle results of outer wall section of untreated bamboo was observed with the ones in smoked and dried bamboo. Statistically significant difference (*p* < 0.05) was observed in the contact angle results between the inner and outer wall sections of all treated and untreated specimens, except in dyed bamboo.

### 3.5. Hardness Test Results

**Figure 10:**
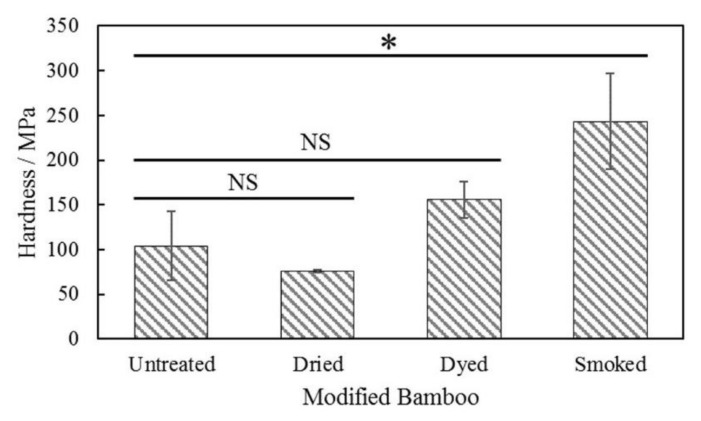
Hardness evaluation of longitudinal-radial section of untreated and modified bamboo specimens (* statistically significant difference, NS = no significant difference).

The hardness of bamboo specimens, measured from the longitudinal-radial section, was found to range between 75 and 250 MPa as indicated on the bar chart of Figure 10. Among the measured specimens, the hardness in smoked bamboo was found to be two-fold higher as compared to the untreated ones. The increase in hardness is attributed due to change in chemical structure resulting from treatment modification.

A summary of the statistical test in terms of an unpaired *t*-test with a significance level of 0.05, as shown in Table 4, was deduced to verify the significance of difference prevailing between tested specimens. Statistically significant difference (*p* < 0.05) was only observed between the hardness test results of untreated and smoked bamboo specimens.

## 4. Discussion

The change in chemical constituent of bamboo specimens due to treatment modification was revealed by Raman analysis. Raman band ratios with respect to normalized peak intensity of 1173 cm^−1^ was found to be a useful indicator to assess the relative change due to treatment modification in bamboo. Smoked treatment was found to affect the intensities of specific peaks as indicated by the ratios of I_1600_/I_1173_ and I_1632_/I_1173_. Significant decrease in the band at 1630 cm^−1^ corresponding to lignin in bamboo [19], was observed in smoked bamboo which was also in accordance to the decreasing trend observed in G band of graphitic carbon [14,15,16]. Hence, this observation clearly indicates the alteration to lignin component in bamboo by thermal effect during smoke treatment, which in turn affected its physical and mechanical properties [5,26].

As observed from FTIR results of Figure 4 and Figure 5 in the range of 400–1800 cm^−1^, significant changes to the main chemical constituents of bamboo mainly took place in its outermost layer. Notable increase in intensities of treated specimens at peak 1114 cm^−1^, which is attributed to lignin component in bamboo, occurred as a result of the thermal effect during the preliminary drying process. The thermal effect above a temperature of 100 °C led to an increase in lignin content via poly-condensation reactions [5,7]. The increase in lignin improves the overall strength of bamboo structure as it accounts for about 20% of the cellular constituent in bamboo [36]. The increase in strength, in particular hardness, along with increasing hydrophobicity, contributes to an increasingly brittle characteristic in smoked bamboo [37,38]. The elevated temperature in smoke treatment at 150 °C is assumed to lead to the degradation of hemicellulose and increase in its hydrophobic nature [7,39]. However, the reduction in hygroscopicity due to the formation of new alcohols and esters linked to lignin [22] was counteracted by the increase in O-H functional groups in the outermost layers which were subjected to steam component of smoke treatment.

The effect of heat during treatment modification is assumed to lead to degradation in the hydrophobic and outermost waxy layer, hence affecting its water-repelling ability, which was slightly decreased as evidenced by the contact angle result in Figure 9. Even though the effect was accentuated in smoked bamboo, marked changes were observed in its outermost layers, whereby the contact angle was found to exceed the innermost one. The degradation of outermost layer was further evidenced by the mean roughness parameter Ra which was found to significantly exceed than that of the untreated one. According to Wenzel, the roughness of a surface with lower hydrophobicity (contact angle < 90°), has a tendency to decrease the contact angle [40,41,42]. This phenomenon was observed in the outermost layer of smoked bamboo as the increase in its roughness led to a decrease in its contact angle, hence confirming a further reduction in its hydrophobic characteristics.

Changes in the physical nature of bamboo affected its mechanical properties as evidenced by the hardness test results. The average hardness value observed in smoke-treated specimen was found to significantly exceed the ones observed in untreated specimen. The increase in hardness, namely in the outermost surface of smoked bamboo is assumed to be the major cause of fracture by longitudinal split in heat-treated bamboo culm as a result of their brittle characteristics.

### Future Recommendation

From this study, non-invasive technique of Raman and FTIR spectroscopy, was found to be ideal to monitor the actual state of modified natural materials like bamboo in service condition. A characterization technique based on Raman spectroscopy could be established in relation to factors affecting durability, namely treatment modification, in order to estimate the product service lifetime and improve other techniques such as preventive maintenance. One challenge that needs to be addressed is maintaining consistency in results obtained due to inherent variability observed in natural materials.

## 5. Conclusions

Like any other natural material, degradation of bamboo over time has direct consequences on its structural capacity. In this study, several proven treatments, commonly used to enhance the dimensional stability of bamboo, were further investigated by spectroscopic techniques in order to probe further into their corresponding effect on the hygroscopic nature of bamboo. First, the molecular footprint of bamboo, obtained by Raman spectroscopic techniques from its radial-tangential section, were instrumental to assess the qualitative changes to the structure following treatment modification. Smoked bamboo, which were subjected to a partial carbonization treatment, displayed similar Raman results as observed in fully carbonized bamboo based on a decreasing trend in the intensities corresponding to the two main D and G bands of graphitic carbon. From FTIR results, the discrepancy in the chemical constituents of bamboo between its innermost and outermost layers could be clearly discerned, especially during treatment modification, as a result of its inhomogeneous structure. The reduction in hygroscopicity in the outermost layers due thermal effect of the treatment, which also led to an increase in lignin content via poly-condensation reactions, was counteracted by the steam component present in smoke treatment.

Finally, the spectroscopy results were validated by physical and mechanical tests of contact angle analysis and hardness test. Marked changes were observed in smoke-treated bamboo as the contact angle of the outermost layers, which significantly exceeded the innermost layers, differed from the untreated ones. Besides, changes in the chemical constituent of smoked bamboo, namely lignin, was evidenced through hardness analysis whereby a net and statistically significant increase relative to untreated bamboo was observed. This study has shown that the non-invasive techniques of spectroscopy analysis can provide accurate estimation of the changes in chemical constituents of bamboo material which correlated well with the observed physical and mechanical characteristics. Given the recognized effect of moisture content on the mechanical characteristics of hygroscopic material like bamboo, recently made available in situ spectroscopic techniques such as portable Raman and FTIR techniques, could be considered to monitor the real-time degradation of such natural materials in service condition.

## Figures and Tables

**Table 1:** Ratio of normalized heights of the absorption peaks of control and modified bamboo.

Relative Intensities against Peak at 1173 cm^−1^	Untreated	Dried	Dyed	Smoked
I_1600_/I_1173_	3.05	3.63	6.46	2.31
I_1632_/I_1173_	1.21	1.42	1.78	0.76

**Table 2:** Characteristic bands of FTIR spectra in the frequency interval of 400 to 1800 cm^−1^ and 2800 to 3800 cm^−1^ obtained from the outside and inside surfaces of bamboo samples.

Peak	Assignment	Frequency (cm^−1^)	References
1	Bending vibration of β-glucosamine bond in cellulose	898	[26]
2	C-O, C-H Primary alcohol, guaiacyl(lignin)	1051	[27]
3	C-H Guaiacyl and syringyl (lignin)	1114	[22]
4	C-O-C Carbohydrate	1159	[28]
5	Guaiacyl ring breathing with CO-stretching (lignin and hemicelluloses), esters	1240	[29,30,31]
6	C=O Quinines and quinine methides, adsorbed water	1657	[23]
7	C=O Carbonyl groups in lignin	1737	[24]
8	C-H in Cellulose and hemicellulose stretching	2925	[25,30,32,33]
9	O-H stretching in Alcohols, phenols, acids, and weakly bounded absorbed water from lignin	3440	[30,32,33,34]
10	O-H stretching of adsorbed water and intermolecular bonded OH	3540	[35]

**Table 3:** Summary of *p*-values from unpaired *t*-test with a significance level of 0.05 to compare the contact angle results of inner and outer wall sections in and between each type of untreated and modified bamboo specimens.

	Group 1				Group 2				*p*-Value (Group 1 & 2)
	Untreated Specimen				Smoked Specimen				
Wall Section	*n*	Mean/°	COV	S.D	*n*	Mean/°	COV	S.D	
**Inner**	3	114.7	0.05	5.74	3	85.3	0.06	5.12	0.0030
**Outer**	3	103.7	0.01	1.04	3	94.3	0.01	0.94	0.0006
***p*-value (Inner & Outer)**	0.0390				0.0370				
					**Dried Specimen**				
**Inner**					3	104.0	0.03	3.12	0.0533
**Outer**					3	91.0	0.04	3.64	0.0069
***p*-value (Inner & Outer)**					0.0108				
					**Dyed Specimen**				
**Inner**					3	96.0	0.12	11.5	0.0716
**Outer**					3	100.7	0.06	6.04	0.4273
***p*-value (Inner & Outer)**					0.5703				

**Table 4:** Summary of *p*-values from unpaired *t*-test with a significance level of 0.05 to compare hardness test results between untreated and modified bamboo specimens.

	Untreated Specimen			Smoked Specimen			*p*-Value
	*n*	Mean	COV	*n*	Mean	COV	
**Hardness (MPa)**	3	104.0	0.37	3	243.4	0.22	0.0219
				**Dried Specimen**			
**Hardness (MPa)**				3	76.0	0.02	0.2778
				**Dyed Specimen**			
**Hardness (MPa)**				3	155.6	0.13	0.1087

## Data Availability

The data presented in this study are available on request from the corresponding author. The data are not publicly available due to private restrictions.
